# Comparison of the effects of imputation methods for missing data in predictive modelling of cohort study datasets

**DOI:** 10.1186/s12874-024-02173-x

**Published:** 2024-02-16

**Authors:** JiaHang Li, ShuXia Guo, RuLin Ma, Jia He, XiangHui Zhang, DongSheng Rui, YuSong Ding, Yu Li, LeYao Jian, Jing Cheng, Heng Guo

**Affiliations:** 1 Department of Public Health, Shihezi University School of Medicine, North 2th Road, Shihezi, 832003 Xinjiang China; 2 Key Laboratory for Prevention and Control of Emerging Infectious Diseases and Public Health Security, the Xinjiang Production and Construction Corps, Shihezi, Xinjiang 832000 China

**Keywords:** Missing data, Imputation methods, Cohort study, Cardiovascular disease, Machine learning

## Abstract

**Background:**

Missing data is frequently an inevitable issue in cohort studies and it can adversely affect the study's findings. We assess the effectiveness of eight frequently utilized statistical and machine learning (ML) imputation methods for dealing with missing data in predictive modelling of cohort study datasets. This evaluation is based on real data and predictive models for cardiovascular disease (CVD) risk.

**Methods:**

The data is from a real-world cohort study in Xinjiang, China. It includes personal information, physical examination data, questionnaires, and laboratory biochemical results from 10,164 subjects with a total of 37 variables. Simple imputation (Simple), regression imputation (Regression), expectation-maximization(EM), multiple imputation (MICE) , K nearest neighbor classification (KNN), clustering imputation (Cluster), random forest (RF), and decision tree (Cart) were the chosen imputation methods. Root Mean Square Error (RMSE) and Mean Absolute Error (MAE) are utilised to assess the performance of different methods for missing data imputation at a missing rate of 20%. The datasets processed with different missing data imputation methods were employed to construct a CVD risk prediction model utilizing the support vector machine (SVM). The predictive performance was then compared using the area under the curve (AUC).

**Results:**

The most effective imputation results were attained by KNN (MAE: 0.2032, RMSE: 0.7438, AUC: 0.730, CI: 0.719-0.741) and RF (MAE: 0.3944, RMSE: 1.4866, AUC: 0.777, CI: 0.769-0.785). The subsequent best performances were achieved by EM, Cart, and MICE, while Simple, Regression, and Cluster attained the worst performances. The CVD risk prediction model was constructed using the complete data (AUC:0.804, CI:0.796-0.812) in comparison with all other models with *p*<0.05.

**Conclusion:**

KNN and RF exhibit superior performance and are more adept at imputing missing data in predictive modelling of cohort study datasets.

**Supplementary Information:**

The online version contains supplementary material available at 10.1186/s12874-024-02173-x.

## Introduction

Cohort studies unavoidably encounter the issue of missing data throughout the investigation [1]. Owing to the study's length, it is possible that some participants may withdraw, lose contact, or miss scheduled visits, resulting in the absence of data. This meaningful but unobtainable data is typically genuine but inaccessible due to some reason [2]. The existence of missing data not only lessens test accuracy and intensifies the intricacy of analysis tasks, but also lowers the effectiveness of statistical work and detrimentally influences data analysis [3]. In a study that analysed how ML prediction models deal with missing data, 56 out of 152 prediction models did not account for any missing data [4]. The inferred conclusions obtained will lose credibility if we are unable to deal with missing values with due care. Therefore, the matter of how to deal with missing data is a critical issue.

The processing of missing data is frequently separated into deletion and imputation [5]. Deletion is the most user-friendly method. The most common method of deletion in medical research is complete case analysis (CCA), which involves deleting cases that contain missing data [4]. The use of this approach may lead to biased results of research. Additionally, removing data can result in a significant loss of meaningful information in datasets with a large sample size [6]. The optimal approach to dealing with missing data is to mitigate its impact on the Institute, rather than merely deleting cases containing missing data. Another method of handling missing data is to fill it in by choosing a suitable value to replace the missing data's value [7]. Several imputation methods have been proposed in studies, but few studies have given guidance on how to use these imputation methods for missing data. No definitive imputation method is appropriate for all cohort study data, and not all the optimal imputation methods applied in various studies are similar. Therefore, it is crucial to examine the methods of imputing missing data in cohort studies for constructing models that predict diseases [4, 8–10].

Cardiovascular disease (CVD) is a frequently occurring chronic non-transmissible ailment that accounts for 31% of worldwide deaths. The occurrence of CVD is progressively rising worldwide. Consequently, CVD is currently the principal reason for mortality and the foremost cause of impairment-adjusted life-year loss globally [11]. The World Health Organization (WHO) estimates that 75% of cardiovascular diseases (CVDs) can be prevented in advance. Therefore, it is necessary to screen the risk factors based on the complex relationships of CVD data to reduce the burden of disease on individuals [12].

This study selected eight statistical and machine learning (ML) methods to impute missing data from a real dataset of a cohort study on cardiovascular disease in Southern Xinjiang, China. The datasets imputed by these imputation methods were compared using performance metrics against the actual data. The ML method was used to build a CVD risk prediction model based on the dataset processed with various missing data imputation methods. The impact of different missing data imputation methods on outcome prediction was then evaluated. The comparison of the two aspects leads to the selection of the most appropriate method for imputation of missing data in the cohort study dataset, which helps in the construction of predictive models. Our research provides references and recommendations for other researchers to choose the appropriate methods when dealing with missing data in predictive modelling of cohort study datasets.

## Methods and materials

### Missing data mechanisms

Before commencing analysis of missing data in a database, an understanding of its mechanism is essential. According to Rubin’s (1976) theory, there are three different mechanisms of missing data: these are known Missing Completely At Random (MCAR), Missing At Random (MAR), and Missing Not At Random (MNAR) [13]. MCAR indicates that the missing data is not dependent on other data and that the missing data is independent of the value of any other data. MAR means that the missing data is related to the data that needs to be collected and is not directly associated with the missing values themselves. MNAR indicates that missing data is not only related to the collected data and is also affected by the missing data itself.

In practical terms, it is difficult to obtain unbiased estimates for MCAR data, which are independent of the data. For the MNAR data, these depend not only on the observed data, but also on the missing data. It was very difficult to use imputation for data on the MNAR mechanism because data not available in the study could not be analysed. Previous research makes it challenging to distinguish these three distinct missing data mechanisms in practice. Therefore, most techniques for handling missing data are based on the MAR data mechanism [2, 14]. Diverse assumptions regarding data missing mechanisms may affect the performance of data imputation methods. Data collected in cohort studies produce associations between variables depending on the characteristics of the participants, producing both MAR and MNAR type data. The imputation of MNAR type data introduces uncertainty in the results because the values of the missing data cannot be accurately estimated. Therefore, in this article, all data in the databases used are defined as MAR missing data, in order to better compare different methods of imputing missing data.

### Imputation method of missing data

Imputation methods for handling missing data predict and replace missing values based on the valid values in other data. The subsequent section briefly details eight frequently used missing data methods selected from statistical and ML methods in this research.

1. Simple imputation (Simple): Substitute every missing data with a quantitative or qualitative attribute of the non-missing part of the data. Typical methods involve substituting the mean for continuous variables and the plural for categorical variables. Simple imputation is an uncomplicated method prevalently commonly used in studies [15]. However, in datasets with complex relationships, this method may produce poor results.
2. Regression imputation (Regression): Regression imputation involves developing regression equations from the complete data in the dataset and employing these equations to anticipate the missing data. The missing data are imputed using the predicted values calculated from the regression equations [16].
3. Expectation–maximization (EM): EM is a method of iteration over missing data based on the criterion of expectation maximization, in which the value of the missing data is estimated based on the complete data already given, and then the missing data is estimated based on the estimated missing data plus the previously observed data. The iteration is divided into two steps, with the first calculating the expectation and the second maximizing it. These two steps are repeated until the method converges [17].
4. Multiple imputation (MICE): MICE is a statistical method for estimating missing data. First, MICE generates several different complete datasets by simulating each missing value multiple times to reflect the uncertainty in the missing values. Then each complete dataset is analysed using the same statistical methods, and finally the results from each dataset are aggregated to produce a final prediction of the missing value imputation. MICE can use various algorithms to impute data, but multiple imputation using chained equations is by far the most common. Currently, multiple imputation is the most frequently used statistical method for imputing missing values [18].
5. K nearest neighbour classification (KNN): The KNN algorithm involves identifying k similar samples by calculating the distance between the complete information of the sample with the missing data and the information of the other samples. The missing data is then estimated using the data from these k samples. The focus of the KNN method is to choose the appropriate distance criterion according to the different types of data, Minkowski distance, Manhattan distance, Hamming distance and Euclidean distance, etc., which are used in different KNN imputation. Currently, the most commonly used methods are based on the Euclidean distance is the most commonly used method for KNN imputation [19].
6. Clustering imputation (Cluster): Clustering imputation by first clustering the complete data in the missing dataset for classification, then dividing the missing data objects into the most similar clusters using a similarity measure and then imputing in with information from within these clusters [20].
7. Decision tree (Cart): Cart is creating a decision tree from the full dataset. The tree is then progressively branched to inseparable using feature conditions. Finally, the missing data is inputted using the corresponding tree. The final prediction is derived from the decision tree by processing continuous and categorical values and dividing the data into two nodes by minimising the variance of the results within each small node, gradually adjusting to the stopping point of the optimal parameters [21].
8. Random forest (RF): RF extracts multiple subsamples from the full data using bootstrap sampling and random feature selection. It builds a tree model for each subsample, and aggregates and combines the individual decision trees into a random forest model. Finally, the missing data set is substituted into the random forest model for processing the missing data [22].

### Study database

This study's dataset is sourced from a cohort study of cardiovascular disease in Southern Xinjiang's population, Chin. Between 2016 and 2022, 12,813 Uyghur residents, aged over 18 and living for more than six months, joined this study. The study collected data on 38 variables from five areas, including personal information, physical examination data, questionnaires, laboratory biochemical results, and outcome indicators. Table 1 lists the complete range of variables.

**Table 1:** List of variables in the dataset

Variable	Variable type	Variable	Variable type
Personal Information			
ID	Classification	Age	Continuous
SEX	Classification		
Physical Examination Data			
Systolic blood pressure(SBP)	Continuous	Diastolic blood pressure(DBP)	Continuous
Height	Continuous	Weight	Continuous
Waistline	Continuous	Hipline	Continuous
Questionnaire Investigation			
Education level	Classification	Work	Classification
Marital status	Classification	Smoke	Classification
Physical exercise	Classification	FHH	Classification
Drink	Classification	FHDM	Classification
FHCHD	Classification		
Laboratory Biochemical Information			
ALP	Continuous	ALB	Continuous
AST	Continuous	A/G	Continuous
APOA	Continuous	APOB	Continuous
APOA/B	Continuous	GLU	Continuous
CKMB	Continuous	CR	Continuous
GLB	Continuous	GGT	Continuous
ALT	Continuous	HDL	Continuous
LDL	Continuous	TC	Continuous
TG	Continuous	TBIL	Continuous
DBIL	Continuous		
Outcome Indicator			
Outcome	Classification		

*Abbreviations*: *FHH* Family history of hypertension, *FHDM* Family history of diabetes, *FHCHD* Family history of coronary heart disease, ALP, alkaline phosphatase, *ALB* albumin, *AST* Aspartate aminotransferase, A/G Albumin/globulin, *APOA* Apolipoprotein A, *APOB* Apolipoprotein B, *APOA/B* Apolipoprotein A/B, *GLU* glucose, *CKMB* Creatine kinase isoenzyme, *GLB* Globulin, *CR* Serum creatinine, *GGT* Glutamyl transpeptidase, *ALT* Glutamic pyruvic transaminase, *HDL* High-density lipoprotein cholesterol, *LDL* Low-density lipoprotein cholesterol, *TC* Total cholesterol, *TG* Triglycerides, *TBIL* Total bilirubin, *DBIL* Direct bilirubin

To ensure accurate data for the study results, it was crucial to obtain complete information. Thus, to avoid any impact on the results from missing data, the samples with missing data were eliminated from the dataset before the study. The process resulted in a total of 11,028 subjects with complete information. Subjects who had a history of CVD before the baseline were then excluded. The final dataset contained 10,164 complete reports with a mean follow-up of 5.47 years. The flowchart of included subjects is presented in Supplementary Figure 1.

The study was approved by the Ethics Review Committee of the First Affiliated Hospital of Shihezi University School of Medicine (shz20101101). All subjects signed an informed consent form before participating in this study. All experimental protocols involving human subjects adhered to the tenets of the Declaration of Helsinki.

### CVD event ascertainment

In this study, the outcome was the first CVD event, defined as a confirmed diagnosis, hospitalization and death during follow-up due to ischaemic heart disease, coronary heart disease, cerebrovascular disease and related conditions (ICD9: codes 390-495). CVD events were identified using hospital diagnostic records, health insurance, follow-up questionnaire responses, and cause-of-death detection systems. If subjects had multiple CVD events during follow-up, the first CVD event was recorded as the outcome [23].

### Machine learning model

Previous studies have shown that support vector machines(SVM) are superior to other ML models in discriminating and calibrating CVD risk in Xinjiang populations when multiple ML methods are used [23]. Therefore, this study chose to use an SVM approach to build an ML model aiming to predict CVD events and compare different missing data imputation methods by assessing their predictive performance [24].

The remaining 37 variables in the dataset, except for ID, were chosen. The dataset generated after imputation using different missing data imputation methods was equally randomly divided into an 80% training set and a 20% test set, with the training set used for model building and hyperparameter tuning. Ten-fold cross-validation is used for the training set to build the optimal model, and a grid search and Bayesian optimization method are used to tune the hyperparameters. After determining the optimal hyperparameters, the optimized parameters are used to build the prediction model in the test set. Finally, the eight missing data imputation methods are compared by comparing the performance of the predictive models built from the databases after the missing data imputation methods have been processed. Supplementary Figure 2 illustrates the flowchart for the predictive modeling.

### Performance evaluation standard of imputation methods

To compare the performance of the different missing value imputation methods, three widely used metrics were chosen for this study: mean absolute error (MAE), root mean square error (RMSE) and area under the curve (AUC) [5].

1. Mean Absolute Error (MAE): MAE is the average difference between the estimated and true value of a measurement, defined as:

1 $${\text{MAE}}=\frac{1}{m}\sum_{i=1}^{m}\left|{y}_{i}-{\widehat{y}}_{i}\right|$$

2) Root Mean Square Error (RMSE): RMSE is the average standard deviation between the estimated and true values of a measurement, defined as:

2 $${\text{RMSE}}=\sqrt{ \frac{1}{m}\sum_{i-1}^{m}{({y}_{i}-\widehat{{y}_{i}})}^{2}}$$

m is the number of missing data in the dataset, ${y}_{i}$ is the true value, and $\widehat{{y}_{i}}$ is the estimated value. In this study, we first calculate the MAE and RMSE for each variable in the dataset individually and then take the mean of all variables as the MAE and RMSE for that dataset. the lower the value of MAE and RMSE, the smaller the deviation between the estimated and true values.

3) Area under the curve (AUC): AUC is the area under the receiver operating characteristic (ROC) curve. The horizontal coordinate of the ROC curve is the positive rate and the vertical coordinate is the true positive rate. It is often used to evaluate the predictive power of a model.

### Statistical analysis

The R software was used to assign 20 per cent missing to the complete data in the real database. This is because a previous study established that the performance of the filler method is independent of the percentage of missing data in the dataset [25]. This study did not impute all variables in the dataset as missing, except for outcome variables and information obtained through ID. Only physical examination data, questionnaires and laboratory biochemical information were imputed as missing. Nest, the missing data set was imputed with eight imputation methods, then assessed alongside the complete data set to compute the MAE and RMSE. SVM-based CVD risk prediction models were then constructed for the datasets processed using the eight missing data estimation methods and for the complete dataset. The AUC of the models was subsequently calculated. Finally, the results of the two comparisons were combined to select the best-performing missing data imputation method.

Continuous variables were described as mean ± standard deviation (SD) and categorical variables as frequencies and percentages. Comparison of features using Student’s t-test or the Mann–Whitney test for continuous variables where appropriate and chi-square tests for categorical variables. *P* < 0.05 was considered to be statistically significant. All statistical analyses in this study were performed using R statistical software 4.2.

## Results

### Study population

A total of 10,164 individuals were included in this study. Table 2 shows the characteristics of continuous variables for eight missing data imputation methods and the true data. The characteristics of categorical variables are provided in Supplementary Table 1. Supplementary Tables 2 and 3 present the baseline clinical characteristics of CVD patients and non-CVD subjects in the training and test sets.

**Table 2:** Comparison of baseline characteristics between complete data and data processed by 8 missing data imputation methods (Continuous variable data)

**Characteristics**	**ALL**	**Simple**	**Regression**	**EM**	**MICE**	**KNN**	**Cluster**	**RF**	**Cart**
SBP (mmHg)	128.5±19.75	128.69±17.67	128.57±19.74	128.6±18.46	128.62±19.9	128.5±19.15	128.58±19.7	128.55±18.78	128.42±18.43
DBP (mmHg)	75±12.31	75.03±11.05	74.96±12.36	75.04±11.53	75.02±13.33	74.99±11.88	75.07±12.34	75.01±11.63	74.97±11.53
Height (cm)	163.27±8.56	163.22±7.65	163.27±8.53	163.26±7.94	163.21±8.56	163.27±8.33	163.18±8.53	163.27±8.15	163.24±8.07
Weight (kg)	69.49±12.91	69.62±11.5	69.51±12.85	69.5±12.32	69.42±12.9	69.48±12.6	69.44±12.88	69.5±12.46	69.47±12.22
Hipline (cm)	101.35±9.75	101.32±8.77	101.31±9.83	101.31±9.35	101.3±9.72	101.34±9.49	101.27±9.76	101.32±9.4	101.25±9.26
Waistline (cm)	91.74±13.67	91.78±12.14	91.74±13.62	91.74±12.8	91.69±13.69	91.74±13.29	91.61±13.52	91.73±13.09	91.67±12.88
A/G	1.59±0.38	1.58±0.33	1.59±0.37	1.58±0.34	1.58±0.36	1.59±0.36	1.58±0.37	1.59±0.35	1.58±0.34
ALB (g/L)	45.41±4.27	45.44±3.81	45.4±4.31	45.43±3.86	44.44±4.3	45.43±4.11	45.42±4.23	45.44±3.98	45.44±3.9
ALP (U/L)	71.31±22.93	71.26±20.28	71.24±22.87	71.29±20.55	71.5±22.77	71.26±21.99	71.11±22.48	71.33±21.02	71.29±20.44
AST (mmol/L)	24.57±13.5	24.66±12.53	24.67±14.08	24.64±12.72	24.63±13.8	24.57±13.23	24.67±14.33	24.61±12.86	24.59±12.71
APOA (g/L)	1.09±0.23	1.09±0.20	1.09±0.23	1.09±0.21	1.09±0.23	1.09±0.22	1.09±0.23	1.09±0.21	1.09±0.21
APOB (g/L)	0.95±0.26	0.95±0.23	0.95±0.26	0.95±0.25	0.95±0.26	0.95±0.25	0.95±0.26	0.95±0.25	0.95±0.25
APOA/B	1.24±0.47	1.23±0.42	1.23±0.47	1.24±0.44	1.24±0.47	1.23±0.46	1.23±0.46	1.24±0.45	1.23±0.43
GLU (mmol/L)	5.03±2.1	5.03±1.88	5.02±2.09	5.03±1.9	5.03±2.15	5.03±2.01	5.04±2.19	5.03±1.94	5.03±1.90
CKMB (ng/L)	19.46±21.15	19.45±18.99	19.37±20.83	19.48±19.09	19.4±20.99	19.44±20.34	19.4±21.01	19.49±19.62	19.44±19.06
CR (μmol/L)	71.55±16.35	71.50±13.98	71.51±16.36	71.53±14.26	71.47±15.87	71.54±15.48	71.47±15.65	71.53±14.72	71.52±14.28
GLB (g/L)	29.67±5.37	29.69±4.79	29.70±5.33	29.69±4.94	29.69±5.37	29.67±5.18	29.67±5.25	29.67±5.05	29.68±4.96
GGT (U/L)	18.74±16.67	18.76±14.90	18.67±16.42	18.73±15.29	18.8±16.78	18.69±16.08	18.71±16.45	18.77±15.53	18.7±15.17
ALT (mmol/L)	28.95±20.93	28.88±18.97	28.82±20.86	28.91±19.42	28.73±20.69	28.89±20.41	28.63±20.79	28.92±19.87	28.79±19.46
HDL (mmol/L)	1.58±0.64	1.58±0.56	1.58±0.64	1.58±0.58	1.58±0.64	1.58±0.61	1.58±0.64	1.58±0.59	1.58±0.58
LDL (mmol/L)	2.63±1.16	2.63±1.05	2.64±1.2	2.64±1.09	2.64±1.17	2.63±1.12	2.64±1.14	2.64±1.09	2.63±1.1
TC (mmol/L)	4.71±1.82	4.72±1.66	4.7±1.76	4.72±1.70	4.7±1.82	4.71±1.79	4.72±1.82	4.72±1.75	4.71±1.71
TG (mmol/L)	1.74±1.4	1.74±1.25	1.74±1.42	1.73±1.28	1.73±1.42	1.73±1.36	1.74±1.4	1.73±1.31	1.73±1.28
TBIL (umol/L)	11.13±9.59	11.10±6.98	11.17±9.35	11.08±7.16	11.06±7.58	11.13±9.41	11.07±7.73	11.09±7.97	11.04±7.19
DBIL (umol/L)	4.54±2.51	4.52±2.23	4.52±2.44	4.53±2.26	4.52±2.5	4.54±2.43	4.54±2.53	4.53±2.32	4.53±2.3

Continuous variable data are expressed as mean ± standard deviation

The mean age of the study population was 38.43 years, with 5,168 men and 4,996 women. During a median follow-up of 5.37 years, 879 subjects were diagnosed with at least one CVD event, with an incidence rate of 8.65%. The data processed by the different methods showed slight variations in values compared with the real data, but there were no significant differences between the characteristics according to the results of the comparison. Patients who developed CVD had higher levels of age and physical examination indicators than non-CVD subjects. Among the laboratory biochemical indicators, CVD patients in the study also had higher variations in ALP, GLU, TC and TG.

### Comparison between imputation data and real data

The performance comparison metrics for all eight missing data imputation methods are shown in Table 3. KNN (MAE 0.2032, RMSE 0.7438) performed best in processing the dataset and achieved the lowest MAE and RMSE. RF (MAE 0.3944, RMSE 1.4866) also performed well. EM (MAE 0.6579, RMSE 2.4929), Cart (MAE 0.7183, RMSE 2.5534) and MICE (MAE 0. 8285, RMSE 2.8699) performed similarly, and the three methods that did not perform well were Simple (MAE 0.8567, RMSE 2.9266), Regression (MAE 1.0235, RMSE 3.5548), Cluster (MAE 1.1383, RMSE 3.8296), and Cluster performing the worst.

**Table 3:** Performance metrics of eight missing data imputation methods for datasets

	**MAE**	**RMSE**	**AUC (95% CI)**
**ALL**			0.804(0.796-0.812)
**Simple**	0.8567	2.9266	0.707*^ (0.695-0.719)
**Regression**	1.0235	3.5548	0.682*^ (0.667-0.697)
**EM**	0.6579	2.4939	0.730*^ (0.719-0.741)
**MICE**	0.8285	2.8699	0.720*^ (0.709-0.731)
**KNN**	0.2032	0.7438	0.769*(0.759-0.779)
**RF**	0.3944	1.4866	0.777*(0.769-0.785)
**CART**	0.7183	2.5534	0.726*^ (0.715-0.737)
**Cluster**	1.1383	1.1383	0.668*^ (0.663-0.683

^*^ indicates *P*<0.05 for AUC vs ALL, ^ indicates *P*<0.05 for AUC vs RF

### Performance comparison of CVD prediction models

Table 3 displays the AUC values for various methods of imputing missing data using SVM to develop a prediction model for Cardiovascular Disease risk. The best results were obtained for the CVD prediction model built using the complete data (AUC: 0.804, CI: 0.796-0.812). Among the methods for imputing missing data, the best discrimination was achieved by the prediction model using RF processed data (AUC: 0.777, CI: 0.769-0.785), which was not significantly different from KNN (AUC: 0.769, CI: 0.759-0.779). Similar predictive power was obtained with the prediction model. In addition, EM (AUC: 0.730, CI:0.719-0.741), Cart (AUC: 0.726, CI:0.715-0.737) and MICE (AUC: 0.720, CI:0.709-0.731) also had a similar predictive model performance. The three methods of Simple (AUC: 0.707, CI: 0.695-0.719), Regression (AUC: 0.682, CI: 0.667-0.697) and Cluster (AUC: 0.668, CI:0.653-0.683) did not perform well in prediction. The AUC of full data was higher than that of RF (*p*<0.05), while there was no significant difference between KNN and RF (*p*=0.436). However, the AUC of RF was higher than that of EM (*p*<0.05).

## Discussion

In this study, eight missing data imputation methods were used to process the missing data in the real-world cohort study dataset, including Simple, EM, Regression, MICE , KNN, Clustering, RF, and Cart. Then, the performance of the eight missing data imputation methods is compared using MAE and RMSE as evaluation metrics. A CVD risk prediction model was also built using SVM. The AUC value was calculated and the effect of different missing data imputation methods on CVD prediction was analysed.

The study indicates that missing data imputation methods do not fully compensate for the impact of missing data on predictive models in cohort study datasets. KNN and RF are found to be more effective in reducing the impact of missing data in the cohort study dataset. Single imputation methods are more based on statistical theory and underestimate the specificity of the sample data, whereas imputation methods based on machine learning frameworks can explore the relationship between the data to a greater extent, achieve better imputation results and provide stronger predictive power. Cohort studies collect data from research populations that include high-dimensional and complex continuous and categorical variables, typically within large sample sizes and multivariate characteristics. But Simple, although easy to operate, it does not take into account the specificity of the data. Using single data to deal with missing data not only artificially alters the distribution of the data, but also underestimates the variance and ignores the correlation between variables, which is not appropriate for such complex data [26].

Both Regression and MICE are based on the construction of regression models to deal with missing data. MICE is currently a commonly used method, but it did not achieve satisfactory results in this study. The dataset in this study was imputed using MICE based on the chaining method and five iterations were run on the dataset. In the regression model, a new regression model is simulated based on the non-missing variables that are used to process the missing values. The accuracy of the regression model used to impute the data will greatly affect the results of Regression imputation. Factors such as the correlation between variables and covariance between variables need to be considered in the study [27]. If you choose to use Regression or MICE in your data processing, it is recommended to build a separate regression model for each variable based on the relationship between the variables to achieve the best performance. According to previous research, MICE is not the best method for imputing missing data, showing that what is most widely used is not necessarily the best [10, 28]. MICE includes many basic imputation methods that impute missing data multiple times but do not always give satisfactory imputation results in cohort studies with high missing rates and uncertain linear relationships [29]. Therefore, when choosing an imputation method for missing data, it is important to make a choice based on information such as the type of data and the degree of missing data. Researchers need to choose an imputation method that is appropriate for the current data, rather than blindly following the trend.

EM, Cluster and KNN performed very differently in this study, with all 3 methods dealing with missing data by using the values of known data in the dataset. As in previous studies, Cluster did not work well in the Cohort Study data set [9]. Cluster focuses on classification, and dividing the data in a dataset into clusters should be the focus of research. If the dataset contains too many samples with missing data, good clustering results cannot be achieved. There is no single standard for clustering. So Cluster is not recommended if high clustering accuracy cannot be achieved with cohort study datasets. According to previous studies, EM performs best in small samples with less than 10% missing data, and in this study, EM also achieved good results [30]. EM iteratively replaces missing data with estimates based on the empirical mean and variance matrix observed in the data. However, EM requires estimation for each missing value and multiple iterations to achieve the best results, a difficult task for large sample datasets [31]. KNN, which has a strong performance record, has also been recommended in previous studies for dealing with missing data [8, 25, 32]. KNN is good at imputing categorical and continuous variables and finds similar data in the dataset to deal with missing data without building a separate model [32]. A large number of samples in cohort research datasets provide a good basis for KNN imputation, and the imputation of missing data based on similar data can also provide a good basis for subsequent prediction or other research. Therefore, KNN is an excellent method for dealing with missing data in cohort studies.

In recent years, ML has been widely studied for its excellent performance in data mining. Imputation methods based on ML can make fuller use of the imputed information for imputation and achieve high estimation accuracy [5, 8]. In this study, all three missing data imputation methods based on ML, KNN, RF and Cart achieved good imputation results, except for Cluster. RF and CART, two imputation methods based on the construction of decision trees, have a high classification accuracy independent of the type of data. They can make imputation data random and uncertain and are more suitable for imputing high-dimensional data [33]. Currently, ML imputation methods have low learning costs, so researchers are encouraged to experiment more with ML methods for missing data.

Our study also has some limitations. The study only selected eight methods for imputing missing data and did not evaluate other methods (Hot‑deck imputation, Neural networks imputation, etc.) or some improvements based on the basic methods, which might have led to different conclusions in other studies. In addition, the AUC values obtained by building the model in this study were not very high and the predictive model did not achieve the best performance because the study did not select some synthetic indicators to include in the model and used all variables to build the predictive model because each variable was considered to contain missing data. In other studies, the predictor variables included in the model should be fully considered in the process of building the model. Finally, about the data, the study uses a dataset from a cohort study, assumes that the pattern of missing data is random and that the dataset contains both continuous and discrete variables. When choosing methods for imputing missing data in other studies, attention should also be paid to information on sample size, patterns of missing data, and types of data in the dataset.

## Conclusions

There can be diverse effects of various methods of imputing missing data in a dataset of cohort study. KNN and RF exhibit superior performance and are more adept at imputing missing data in cardiovascular cohort study datasets. However, it is important to note that real data cannot be replaced. Therefore, developing a robust experimental plan and optimizing activities to minimize missing data is the optimal method.

### Supplementary Information

**Additional file 1:** **Supplementary Figure 1.** Flow diagram of subjects included. **Supplementary Figure 2.** Flow diagram of CVD risk prediction model building process. **Supplementary Table 1.** Comparison of baseline characteristics between complete data and data processed by 8 missing data interpolation methods (Categorical data). **Supplementary Table 2**. Comparison of study subjects in the training and test sets (continuous data). **Supplementary Table 3**. Comparison of research objects between training set and test set (Categorical data).

## Data Availability

The datasets used during the current study are available from the corresponding author upon reasonable request. The Chinese questionnaire copy may be requested from the authors.
