# Supplementary material for: Comparison of the effects of imputation methods for missing data in predictive modelling of cohort study datasets
Source: BMC Med Res Methodol. 2024 Feb 16;24:41. doi: 10.1186/s12874-024-02173-x (PMC10870437; doi:10.1186/s12874-024-02173-x)
Supplement: Supplementary file 1 — Additional file 1: Supplementary Figure 1. Flow diagram of subjects included. Supplementary Figure 2. Flow diagram of CVD risk prediction model building process. Supplementary Table 1. Comparison of baseline characteristics between complete data and data processed by 8 missing data interpolation methods (Categorical data). Supplementary Table 2. Comparison of study subjects in the training and test sets (continuous data). Supplementary Table 3. Comparison of research objects between training set and test set (Categorical data). [file 12874_2024_2173_MOESM1_ESM.docx]

Supplementary Material

**
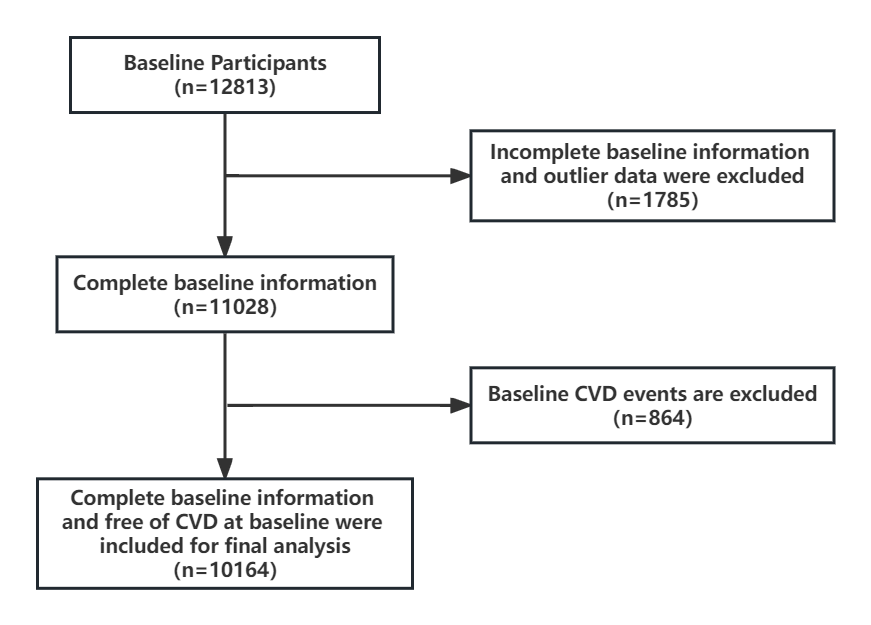
**

**Supplementary Figure 1.** Flow diagram of subjects included .

**
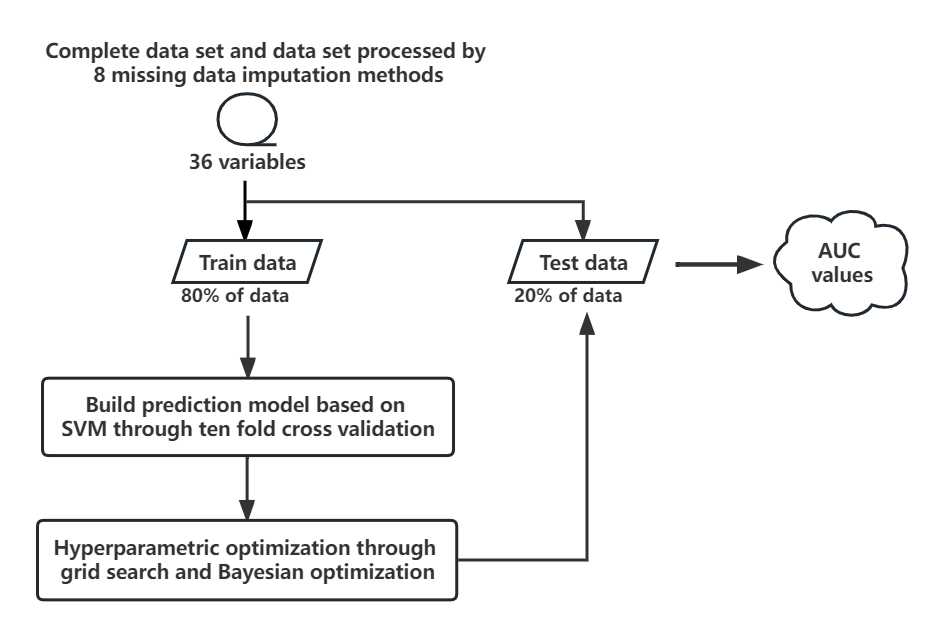
**

**Supplementary Figure 2.** Flow diagram of CVD risk prediction model building process

| **Characte-ristics** |  | **ALL** | **Simple** | **Regression** | **EM** | **MICE** | **KNN** | **Cluster** | **RF** | **CART** |
| --- | --- | --- | --- | --- | --- | --- | --- | --- | --- | --- |
| Education level | Illiteracy | 4275（42.1%） | 3409（33.5%） | 4178（41.2%） | 3563（35.1%） | 4274（42.1%） | 4200（41.3%） | 4277（42.1%） | 3994（39.3%） | 3409  （33.5%） |
|  | Primary school | 2729（26.8%） | 4219（41.5%） | 2845  （28%） | 3819（37.6%） | 2741  （27%） | 2858（28.1%） | 2726（26.8%） | 3162（31.1%） | 4065  （40%） |
|  | Junior high school | 2253  （22.2%） | 1807  （17.8%） | 2237  （22%） | 2034  （20%） | 2252（22.2%） | 2233  （22%） | 2239  （22%） | 2183（21.5%） | 1887  （18.6%） |
|  | High school | 557（5.5%） | 447（4.4%） | 571（5.6%） | 466（4.6%） | 550（5.4%） | 555（5.5%） | 562（5.5%） | 543（5.3%） | 521  （5.1%） |
|  | College degree and above | 350  （3.4%） | 282  （2.8%） | 333  （3.2%） | 282  （2.8%） | 347  （3.4%） | 318  （3.1%） | 360  （3.5%） | 282  （2.8%） | 282  （2.8%） |
| Occupa  -tion | Agriculture and animal husbandry | 8582  （84.4%） | 8912  （87.7%） | 8375  （82.4%） | 8297  （81.6%） | 8585  （84.5%） | 8571（84.3%） | 8587（84.5%） | 8484（83.5%） | 8770  （86.3%） |
|  | Industrials | 187（1.8%） | 151（1.5%） | 469（4.6%） | 764（7.5%） | 191（1.9%） | 214（2.1%） | 190（1.9%） | 395（3.9%） | 151  （1.5%） |
|  | civil servant  Or  Self-employed | 487  （4.8%） | 389  （3.8%） | 472  （4.6%） | 391  （3.8%） | 491  （4.8%） | 592  （5.8%） | 488  （4.8%） | 562  （5.5%） | 531  （5.2%） |
|  | Others | 908（8.9%） | 712（7.0%） | 848（8.4%） | 712  （7%） | 897（8.8%） | 787（7.7%） | 899（8.8%） | 723（7.1%） | 712  （7%） |
| Marital status | Unmarried | 825  （8.1%） | 643  （6.3%） | 804  （7.9%） | 643  （6.3%） | 807  （7.9%） | 819  （8.1%） | 809  （8%） | 768  （7.6%） | 774  （7.6%） |
|  | Married | 8641  （85%） | 8963（88.2%） | 8676（85.4%） | 8962（88.2%） | 8635  （85%） | 8650（85.1%） | 8642  （85%） | 8724（85.8%） | 8832  （86.9%） |
|  | Widow | 369（3.6%） | 293（2.9%） | 359（3.5%） | 294（2.9%） | 379（3.7%） | 399（3.9%） | 379（3.7%） | 407  （4%） | 293  （2.9%） |
|  | Live alone | 329（3.2%） | 265（2.6%） | 325（3.2%） | 265（2.6%） | 343（3.4%） | 296（2.9%） | 334（3.3%） | 265（2.6%） | 265  （2.6%） |
| Physical exercise | Frequently | 1038  （10.2%） | 826  （8.1%） | 1022  （10%） | 826  （8.1%） | 1022（10.1%） | 955  （9.4%） | 1030（10.1%） | 838  （8.2%） | 826  （8.1%） |
|  | Occasionally | 569（5.6%） | 451（4.4%） | 598（5.9%） | 575（5.7%） | 577（5.7%） | 653（6.4%） | 559（5.5%） | 758（7.5%） | 451  （4.4%） |
|  | Rarely | 8557  （84.2%） | 8887（87.4%） | 8544（84.1%） | 8763（86.2%） | 8565（84.3%） | 8556（84.2%） | 8575（84.4%） | 8568（84.3%） | 8887  （87.4%） |
| Smoke | Yes | 1885  （18.5%） | 1518（14.9%） | 1861（18.3%） | 1602（15.8%） | 1881  （18.5%） | 1884（18.5%） | 1909（18.8%） | 1828  （18%） | 1589  （15.6%） |
|  | No | 8279  （81.5%） | 8646（85.1%） | 8303（81.7%） | 8562（84.2%） | 8283  （81.5%） | 8280（81.5%） | 8255（81.2%） | 8336  （82%） | 8575  （84.4%） |
| Drink | Yes | 542（5.3%） | 434（4.3%） | 547（5.4%） | 434（4.3%） | 549（5.4%） | 542（5.3%） | 544（5.4%） | 519（5.1%） | 434  （4.3%） |
|  | No | 9233  （90.8%） | 9730（95.7%） | 9617（94.6%） | 9730（95.7%） | 9615（94.6%） | 9622（94.7%） | 9620（94.4%） | 9645（94.9%） | 9730  （95.7%） |
| FHH | Yes | 931（9.2%） | 739（7.3%） | 923（9.1%） | 750（7.4%） | 936（9.2%） | 925（9.1%） | 927（9.1%） | 843（8.3%） | 739  （7.3%） |
|  | No | 9233  （90.8%） | 9425（92.7%） | 9241（90.9%） | 9414（92.6%） | 9228（90.8%） | 9239（90.9%） | 9237（90.0%） | 9321（91.7%） | 9425  （92.7%） |
| FHDM | Yes | 387（3.8%） | 319（3.1%） | 396（3.9%） | 319（3.1%） | 407  （4%） | 383（3.8%） | 405  （4%） | 343（3.4%） | 319  （3.1%） |
|  | No | 9777  （96.2%） | 9845（96.9%） | 9768（96.1%） | 9845（96.9%） | 9757  （96%） | 9781（96.2%） | 9769  （96%） | 9821（96.6%） | 9845  （96.9%） |
| FHCHD | Yes | 592（5.8%） | 483（4.8%） | 600（5.9%） | 483（4.8%） | 591（5.9%） | 589（5.8%） | 588（5.8%） | 524（5.2%） | 483  （4.8%） |
|  | No | 9572  （94.2%） | 9681（95.2%） | 9564（94.1%） | 9671（95.2%） | 9572（94.2%） | 9575（94.2%） | 9576（94.2%） | 9640（94.8%） | 9681  （95.2%） |

**Supplementary Table 1.**

Comparison of baseline characteristics between complete data and data processed by 8 missing data interpolation methods (Categorical data)

Categorical variable data are expressed as n(%)

| **Characteristics** | **Training Set** | | | **Test Set** | | |
| --- | --- | --- | --- | --- | --- | --- |
|  | **Non-CVD**  **(n=7419)** | **CVD (n=712)** | **P**  **value** | **Non-CVD**  **(n=1866)** | **CVD (n=167)** | **P**  **value** |
| Age, (years) | 37.21±12.71 | 50.71±12.88 | <0.01 | 37.43±12.74 | 51.30±13.18 | <0.01 |
| SBP(mmHg) | 127.16±18.49 | 143.0±26.60 | <0.01 | 127.18±18.46 | 141.93±23.91 | <0.01 |
| DBP(mmHg) | 74.45±11.94 | 81.08±15.04 | <0.01 | 74.37±11.74 | 80.4±13.61 | <0.01 |
| Height(cm) | 163.54±8.53 | 160.09±8.33 | <0.01 | 163.70±8.43 | 160.17±9.04 | <0.01 |
| Weight(kg) | 69.13±12.83 | 72.49±12.78 | <0.01 | 69.37±12.84 | 73.68±14.64 | <0.01 |
| Hipline(cm) | 101.03±9.68 | 105.01±9.59 | <0.01 | 100.93±9.63 | 104.46±11.15 | <0.01 |
| Waistline(cm) | 91.13±13.48 | 98.13±14.59 | <0.01 | 69.37±12.84 | 97.04±15.70 | <0.01 |
| A/G | 1.60±0.37 | 1.50±0.31 | <0.01 | 1.59±1.57 | 1.54±0.49 | 0.09 |
| ALB(g/L) | 45.52±4.14 | 44.76±3.89 | <0.01 | 45.33±4.74 | 44.29±5.49 | <0.01 |
| ALP(U/L) | 70.96±22.98 | 74.55±24.05 | <0.01 | 71.23±22.15 | 75.64±22.84 | 0.01 |
| AST(mmol/L) | 24.53±13.53 | 25.24±12.23 | 0.17 | 24.53±13.87 | 24.30±8.63 | 0.84 |
| APOA(g/L) | 1.09±0.23 | 1.09±0.23 | 0.76 | 1.08±0.24 | 1.10±0.23 | 0.12 |
| APOB(g/L) | 0.94±0.26 | 1.04±0.26 | <0.01 | 0.94±0.26 | 1.06±0.29 | <0.01 |
| APOA/B | 1.24±0.46 | 1.11±0.23 | <0.01 | 1.26±0.53 | 1.08±0.47 | <0.01 |
| GLU(mmol/L) | 5.01±2,04 | 5.47±2.74 | <0.01 | 4.97±2.00 | 5.19±2.06 | 0.18 |
| CKMB(ng/L) | 19.53±21.17 | 19.12±21.49 | 0.62 | 19.47±21.27 | 17.48±17.27 | 0.24 |
| CR(μmol/L) | 71.45±15.99 | 71.32±17.46 | 0.85 | 72.23±17.39 | 69.05±15.09 | 0.02 |
| GLB(g/L) | 29.58±5.36 | 30.64±5.27 | <0.01 | 29.59±5.37 | 30.18±5.51 | 0.17 |
| GGT(U/L) | 18.51±16.56 | 20.88±17.60 | <0.01 | 18.52±16.42 | 22.08±19.01 | <0.01 |
| ALT(mmol/L) | 28.95±20.43 | 29.73±28.94 | 0.48 | 28.64±19.59 | 28.97±16.39 | 0.83 |
| HDL(mmol/L) | 1.59±0.64 | 1.44±1,29 | <0.01 | 1.60±0.66 | 1.50±0.70 | 0.08 |
| LDL(mmol/L) | 2.63±1.24 | 2.77±2.71 | <0.01 | 2.59±0.94 | 2.82±0.81 | <0.01 |
| TC(mmol/L) | 4.72±2.01 | 4.82±1.28 | 0.18 | 4.70±1.10 | 4.97±1.06 | <0.01 |
| TG(mmol/L) | 1.70±1.36 | 1.96±1,31 | <0.01 | 1.77±1.58 | 1.96±1.53 | 0.13 |
| TBIL(umol/L) | 11.18±10.32 | 11.13±6.43 | 0.89 | 10.98±7.69 | 10.63±5.02 | 0.57 |
| DBIL(umol/L) | 4.56±2.55 | 4.51±2.98 | 0.60 | 4.49±1.17 | 4.34±2.00 | 0.40 |

**Supplementary Table 2**

Comparison of study subjects in the training and test sets (continuous data).

Continuous variable data are expressed as mean ± standard deviation

| Characteristics |  | Training Set | | | Test Set | | |
| --- | --- | --- | --- | --- | --- | --- | --- |
|  |  | Non-CVD  (n=7419) | CVD (n=712) | P  value | Non-CVD  (n=1866) | CVD (n=167) | P  value |
| Sex | Male | 3854（51.9%） | 258（36.2%） | <0.01 | 990(53.1%) | 66(39.5%) | <0.01 |
|  | Female | 3565（48.1%） | 454（63.8%） |  | 879(46.9%) | 101(60.5%) |  |
| Education level | Illiteracy | 3023（40.7%） | 401（56.3%） | <0.01 | 752(40.3%) | 99(59.3%) | <0.01 |
|  | Primary school | 2029（27.3%） | 155（21.8%） |  | 505(27.1%) | 40(24.0%) |  |
|  | Junior high school | 1694（22.8%） | 98（13.8%） |  | 446(23.9%) | 15(9.0%) |  |
|  | High school | 410（5.5%） | 23（3.2%） |  | 118(6.3%) | 6(3.6%) |  |
|  | College degree and above | 263（3.5%） | 35（4.9%） |  | 45(2.4%) | 7(4.2%) |  |
| Occupation | Agriculture and animal husbandry | 6271（84.5%） | 593（83.3%） | 0.11 | 1583(84.8%) | 135(80.8%) | 0.46 |
|  | Industrials | 149（2.0%） | 8（1.1%） |  | 28(1.5%) | 2(1.2%) |  |
|  | civil servant  Or  Self-employed | 357（2.8%） | 45（6.3%） |  | 75(4.0%) | 10(6%) |  |
|  | Others | 642（3.3%） | 66（9.3%） |  | 180(9.6%) | 20(12%) |  |
| Marital status | Unmarried | 665（9.0%） | 16（2.2%） | <0.01 | 141(7.6%) | 3(1.8%) | <0.01 |
|  | Married | 630（84.9%） | 610（85.7%） |  | 1587(85.0%) | 142(85.0%) |  |
|  | Widow | 210（2.8%） | 72（10.1%） |  | 71(3.8%) | 16(9.6%) |  |
|  | Live alone | 242（3.3%） | 14（2.0%） |  | 67(3.6%) | 6(3.6%) |  |
| Physical exercise | Frequently | 740（10.0%） | 60（8.4%） | 0.16 | 222(11.9%) | 16(9.6%) | 0.52 |
|  | Occasionally | 417（5.6%） | 32（4.5%） |  | 112(6.0%) | 8(4.8%) |  |
|  | Rarely | 6262（84.4%） | 620（87.1%） |  | 1532(82.1%) | 143(85.6%) |  |
| Smoke | Yes | 1439（19.4%） | 67（9.4%） | <0.01 | 361(19.3%) | 21(12.6%) | 0.03 |
|  | No | 5983（80.6%） | 645（90.6%） |  | 1505(80.7%) | 146(87.4%) |  |
| Drink | Yes | 407（5.5%） | 22（3.1%） | <0.01 | 106(5.7%) | 7(4.2%) | 0.42 |
|  | No | 7012（94.5%） | 690（96.9%） |  | 1760(94.3%) | 160(95.8%) |  |
| FHH | Yes | 674（9.1%） | 63（8.8%） | 0.83 | 177(9.5%) | 17(10.2%) | 0.77 |
|  | No | 6745（90.9%） | 649（91.2%） |  | 1689(90.5%) | 150(89.8%) |  |
| FHDM | Yes | 279（3.8%） | 31（4.4%） | 0.43 | 74(4.0%) | 3(1.8%) | 0.16 |
|  | No | 7140（96.2%） | 681（95.6%） |  | 1792(96.0%) | 164(98.2%) |  |
| FHCHD | Yes | 431（5.8%） | 39（5.5%） | 0.71 | 114(6.1%) | 8(4.8%) | 0.49 |
|  | No | 6988（94.2%） | 673（94.5%） |  | 1752(93.9%) | 159(95.2%) |  |

**Supplementary: Table 3**

Comparison of research objects between training set and test set (Categorical data)

Categorical variable data are expressed as n(%)
